# Urban connected vehicle lane planning based on improved Frank Wolfe algorithm

**DOI:** 10.1371/journal.pone.0321540

**Published:** 2025-04-22

**Authors:** Anqi Jiang, Faziawati binti Abdul Aziz, Norsidah binti Ujang, Mohd Afzan bin Mohamed

**Affiliations:** 1 Faculty of Design and Architecture, University Putra Malaysia, Serdang Selangor, Malaysia; 2 Faculty of Built Environment, University Malaysia Sarawak (Unimas), Kota Samarahan Sarawak, Malaysia; Southwest Jiaotong University, CHINA

## Abstract

As the new generation of information technology matures and improves, the functions of intelligent connected vehicles become more and more perfect, and the number of urban connected vehicles is also increasing. To provide an effective optimization scheme to the mixed traffic flow road network in the networked environment, the study investigates the lane planning for urban connected vehicles method. First, a lane planning for urban connected vehicles bi-level programming model is constructed. Then, the upper-level model is solved using improved whale optimization, and the lower-level model is solved using improved Frank-Wolfe algorithm. The results showed that the accuracy and recall of the proposed improved whale optimization algorithm on the Iris dataset were 95.27% and 92.65%, respectively, which were superior to traditional whale optimization algorithm, moth flame optimization algorithm, moth flame optimization algorithm combined with chaos strategy, and adaptive firefly optimization algorithm. The proposed improved Frank Wolfe algorithm can converge at around 30 iterations, with a convergence limit of around 10^-4^, which is superior to the traditional Frank Wolfe algorithm. The minimum total travel cost of the road system gradually decreases with the increase of the fairness index threshold. The experimental results demonstrate the effectiveness of the proposed urban connected vehicle lane planning model and solving algorithm. The research results contribute to improving the operational safety and efficiency of the road network TS, thereby improving the current traffic situation of the urban TS.

## 1. Introduction

With the acceleration of urbanization and the continuous growth of car ownership, urban transportation is facing unprecedented pressure and has become a key factor restricting the sustainable development of cities. Lane planning refers to the construction, planning, and management of road systems based on the current situation and characteristics of a specific transportation system (TS), and plays a crucial role in road traffic design [1]. Intelligent Connected Vehicles (CVs) are an important path for the transformation and upgrading of the automotive industry. However, traditional lane planning methods are often based on static traffic models, which are difficult to adapt to real-time changing traffic flow and fail to fully consider the interaction and collaboration between CVs, resulting in low traffic operation efficiency. Therefore, with the increasing application of CVs in urban transportation, traditional lane planning methods are no longer able to adapt to the complexity and diversity of modern transportation, and research on lane planning has become particularly urgent [2,3]. Therefore, the research aims to explore the optimization plan for urban connected vehicle lane planning, in order to improve the overall traffic efficiency of the city. In an effort to increase the stability and safety of autonomous truck driving, W. Hu et al. separated the driving decision-making process into intention generation and feasibility assessment. The study assessed the aggression index and suggested a probabilistic framework for lane design and decision-making. The outcomes revealed that the suggested framework could successfully deliver truck trajectories and lane change decisions with useful real-world uses [4]. S. Liu et al. developed an optimization framework for guiding bike lane planning from bike trajectories by formalizing the bike lane planning problem based on the utility function of cyclists. The findings suggested that the suggested paradigm had some viability and efficacy and supported the adoption of data-driven urban planning initiatives [5]. For the challenge of autonomous lane changing by self-driving cars in realistic and complex driving scenarios, S. Li et al. proposed an integrated approach based on deep reinforcement learning algorithms with safe action set technology. As per the findings, the suggested approach has the potential to steer the vehicle more precisely, resulting in reduced trip time, increased computing efficiency, and enhanced real-time performance [6]. In an effort to increase driving safety and adjust to road friction and vehicle speed for autonomous driving, J. Hu et al. developed an adaptive lane-change lane planning scheme. They also created a 7th-order polynomial function to achieve trajectory smoothing. According to the findings, the suggested plan might successfully increase lane planning efficiency while maintaining acceptability and traceability [7]. M. Kang et al. addressed the issue of self-driving cars mixing with conventional cars that may lead to traffic accidents by deploying self-driving car-only lanes to address the negative impacts in a 75% market penetration environment. The study also proposed an optimal operational strategy for self-driving car-only lanes for more viable operations from an operational efficiency perspective. The study’s findings may be able to offer some guidelines for managing highways in the age of autonomous vehicles [8]. To attempt to solve the issue of creating dedicated lanes for self-driving cars, Z. Yao et al. used the following automobile model to generate the fundamental mixed traffic flow (TF) diagram. The study analyzed the traffic capacity of dedicated lanes for self-driving cars and discussed the sensitivity of relevant parameters in the basic diagram. The results indicated that the development of dedicated lanes with a reasonable penetration rate (PR) of self-driving cars would not lead to wasted resources and traffic congestion [9]. Although the above studies have analyzed lane planning, W. Hu [4], S. Liu [5], and J. Hu et al. [7] focused on the lane planning problem of traditional traffic flow, and did not consider the mixed traffic of autonomous vehicle and traditional vehicles. M. Kang [8] and Z. Yao et al. [9] considered the issue of mixed traffic flow, but overlooked the problem of traffic fairness.

Lane programming model is an important part of transportation planning, which achieves the best TF strategy and planning scheme through the application of mathematical models. With the rapid development of various intelligent algorithms, it provides efficient and highly accurate solutions for solving lane programming models, which plays a key role in improving transportation efficiency and reducing congestion [10]. The Frank-Wolfe (FW) algorithm is an optimization algorithm for solving linear constrained problems, proposed by Frank and Wolfe in 1956. It is particularly suitable for transforming a nonlinear optimization problem into a series of linear programming problems to solve, and has low computational complexity when dealing with large-scale optimization problems [11]. By merging gradient tracking and Nesterov’s momentum approaches, J. Hou et al. introduced a distributed stochastic FW solver for the effective solution of distributed stochastic optimization problems. The procedure dealt with convex constrained issues by using the FW algorithm. The outcomes demonstrated the quick convergence rate and efficacy of the suggested algorithm in numerical simulations of multiple rival systems [12]. Rinaldi et al. defined short-step chaining procedures that skip the gradient computation in successive short steps. The results indicated that both the FW algorithm and its variants could satisfy the conditions when minimization problems on polyhedra were considered [13]. According to P. Dvurechensky et al., large-scale optimization in computational statistics and machine learning has grown to rely heavily on projection-free optimization using FW algorithm versions. However, projection-free minimization methods had no theoretical convergence guarantees in binary classification. This study therefore developed a FW algorithm with standard provable convergence. The results demonstrated that the proposed algorithm had fast convergence and was able to produce linear convergence [14]. A variant of the FW algorithm was investigated by G. V. Aivazian et al. This variation chose the step size (SS) parameter in an adaptive manner based on the objective function (OF)’s smoothness information. According to the findings, the approach ensured at least a twofold decrease of the function residuals with a quick solution efficiency and was sublinearly convergent in the solution of problems with a convex OF [15]. The issue that the classic FW algorithm could only be used in cases when the feasible domain was bounded was addressed by H. Wang et al. The outcomes showed that the suggested approaches outperformed the others in solving convex optimization problems and enhanced the FW algorithm’s practical usefulness [16]. To solve the issue of recognizing malicious sensors in IoT edge computing systems, J. Sangeethapriya et al. developed an FW supervised machine learning technique that assisted to increase the security of IoT edge servers. According to the findings, the suggested strategy was practical and efficient because it had a high detection accuracy and a low computational cost [17]. Through the above research on the Frank Wolfe algorithm, it can be concluded that the algorithm has been proven to have good performance in solving linear and convex constraint problems. However, its application in lane planning problems is still relatively rare, and most studies have not proposed measures to address the slow convergence speed of the Frank Wolfe algorithm in the later stages of iteration.

In summary, the mixed traffic flow composed of manually driven cars and CVs has changed the composition of traditional traffic flow, bringing new traffic characteristics and uncertainties, which poses new challenges to lane planning methods. In a mixed traffic environment, lane planning for CVs can better organize mixed traffic flow and improve traffic efficiency. However, lane planning for CVs is equivalent to granting privileges to CVs, occupying some of the existing road resources in the TS and causing unfair distribution of traffic benefits to a certain extent. However, most current intelligent connected vehicle lane planning methods overlook the issue of traffic fairness, which may exacerbate traffic inequality and affect the sustainability of the TS. Therefore, the study builds a model of lane planning for urban CVs with the constraints of the construction cost of dedicated lanes and traffic fairness, by considering the situation where CVs are mixed with ordinary manually driven vehicles. The research results not only provide new solutions for traffic management in intelligent connected vehicle environments, but also enable fair allocation of transportation resources, thereby promoting the development of intelligent TS. There are two main innovations in the research. The first point is to consider cost constraints and also propose traffic fairness constraints to build a lane planning model. The second point is to address the problem of Whale Optimization Algorithm (WOA) getting stuck in one end of binary variables in solving lane planning models, and to propose using logical operations to fully search the solution space. The main structure of the study is divided into 3 parts. The first part is to build a lane planning for urban CVs bi-level programming model (BLPM) and propose the improved WOA and FW algorithms to solve the model. The second part analyzes the application effect of the proposed algorithms. The last part summarizes the whole study.

## 2. Methods and materials

The study initially develops a BLPM for lane planning for urban CVs in order to increase the effectiveness of the road network TS in the event of mixed traffic between CVs and regular manually driven vehicles. Then, the improved WOA and Frank Wolfe algorithm are proposed to solve the model.

### 2.1. lane planning for urban CVs modeling

**Fig 1:**
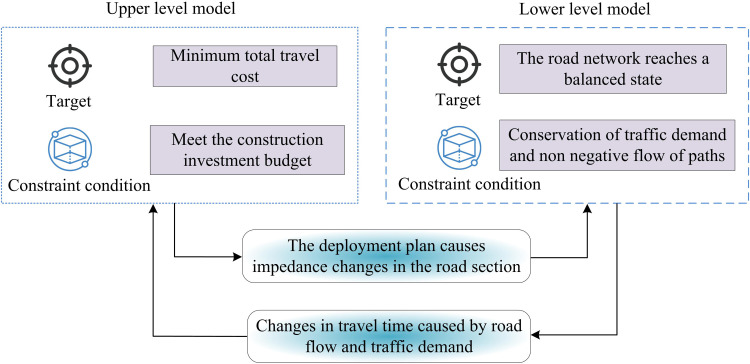
The architecture of the BLPM.

The lane planning for urban CVs problem belongs to the Stackelberg game problem, which is a form of game in a multistage recursive decision system. Therefore, to characterize the two-stage dynamic game, the study solves it by building a BLPM [18]. To facilitate the calculation, the study makes the following four assumptions in conjunction with the actual situation. First, the planning scheme of lanes can meet the demand of CVs, and the CVs will concentrate on the planned lanes. Second, all vehicles on the road are converted to cars, and the travel demand is in terms of passenger car unit (PCU). A PCU is the equivalent traffic volume of a standard vehicle model converted from actual motorized and non-motorized traffic by a certain conversion factor. This is used to measure the influence of different transportation modes on road TF for comparison and analysis. Third, the travel time function of a road section is a continuously derivable function about the TF on the road section. Fourth, travelers are usually able to be accurately informed of the traffic conditions on the roads of the entire road network and make travel choices. To obtain the lane planning for CVs scheme with certain traffic benefits, the study takes the lowest total travel cost (TTC) of the road system as the OF, and the construction cost of the dedicated lanes and the traffic fairness as the constraints. Fig 1 depicts the architecture of the bi-level programming approach.

The global optimization of the entire system is taken into consideration by the upper level programming model (ULPM) in Fig 1, which is primarily in charge of handling high-level decision-making difficulties. The placement of the dedicated lane planning needs to be established while considering the impact of the lower level model. The distribution of TFs within the ULPM’s limitations is the focus of the lower level programming model (LLPM). The study’s planning goal for the ULPM is to minimize the total cost of travel within the TS. Equation (1) provides the calculation of the TS’s total trip cost.


$$\min Z_{y}=\underset{y}{\min}\sum_{rs\in W}\sum_{m\in M}t_{rs}^{m}\eta_{m}$$ (1)

In Equation (1), $Z_{y}$ denotes the TTC of the TS corresponding to scenario *y*. *r* denotes the origin node. *s* denotes the destination. *m* denotes the travel mode. *M* denotes the set of travel modes. $t_{rs}^{m}$ denotes the travel time. $\eta_{m}$ denotes the travel time value (TTV) factor. The TTV coefficient of traditional human-driven vehicles is 3.4 yuan/min, and the TTV coefficient of self-driving vehicles is 2.8 yuan/min. Autonomous vehicle are expected to improve road safety and traffic capacity. The planning of connected vehicle lanes will separate CVs from traditional vehicles, which has potential inequality problems [19,20]. Therefore, in order to reasonably allocate transportation resources and ensure that the impact of roadway planning on the travel benefits of different groups is generally consistent, the study also considers transportation equity. The user transportation equity constraint is shown in Equation (2).


$$e_{y}=\underset{rs\in W}{\max}E_{rs}\leq \alpha$$ (2)

In Equation (2), $e_{y}$ denotes the user transportation fairness indicator corresponding to scheme *y*. $E_{rs}$ denotes the user transportation fairness indicator corresponding to *r* to *s*, and the value range is [0,1]. As $E_{rs}$ gets closer to 0 the fairer it is, and vice versa the less fair it is. *α* denotes the fairness index threshold. The constraint of the construction cost of the dedicated lane for CVs is shown in Equation (3).


$$g_{y}=\sum_{a\in A}y_{a}l_{a}d<B$$ (3)

In Equation (3), $g_{y}$ denotes the construction cost corresponding to the program. $y_{a}$ denotes the binary decision variable for lane setting on road section *a*. A lane is set up if $y_{a}=1$ and vice versa. $l_{a}$ denotes the length of the lane. *d* denotes the construction cost per unit length. *B* denotes the construction budget. Lane allocation can help alleviate traffic congestion without building new roads, and has been proven to be NP hard [21]. In summary, the OF of the ULPM of lane for urban CVs is to minimize the TTC of the TS. Its constraints include transportation equity and construction cost. In the LLPM, the impedance function of each road segment should be determined to solve the traffic distribution problem in the TS, and then the traffic volume on each road is projected. Impedance function refers to the functional relationship between the traveling time of the road segment and the traffic load of the road segment, and it is the basis of traffic network analysis. This helps to analyze and optimize TF and improve traffic efficiency. The Bureau of Pubic Roads (BPR) function is a classical impedance function, which is an impedance function used to calculate the free travel time of a road segment. It determines the correction of traveling time of a road segment by considering the relationship between traveling time and traffic volume of the road segment [22,23]. The BPR function is shown in Equation (4).


$$t_{a}=t_{a}^{z}\left[1+\alpha {\left(\frac{x_{a}}{c_{a}}\right)}^{\beta }\right]$$ (4)

In Equation (4), *α* and *β* denote calibration parameters. $t_{a}^{z}$ denotes the free-flow travel time of section *a*. $c_{a}$ denotes the capacity. $x_{a}$ denotes the flow rate. Equation (4) shows that the capacity of the roadway section affects the BPR function. Therefore, the study derives the basic graphical model of the roadway section through intelligent driver model (IDM), which in turn calculates the capacity. IDM is a kind of following model for simulating the following behavior between vehicles in a TF, which has been widely used in simulating the characteristics of TF and optimizing the design of traffic systems [24,25]. According to the characteristics of steady TF, the mathematical expression of the flow rate can be obtained as shown in Equation (5).


$$q_{e}=\frac{v_{e}}{s_{e}} \{\begin{array}{c} \frac{v_{e}}{s_{e}},s_{e}\geq s_{0}+Tv_{z}\\ \frac{v_{e}}{s_{0}+Tv_{z}},s_{e}<s_{0}+Tv_{z} \end{array}$$ (5)

**Fig 2:**
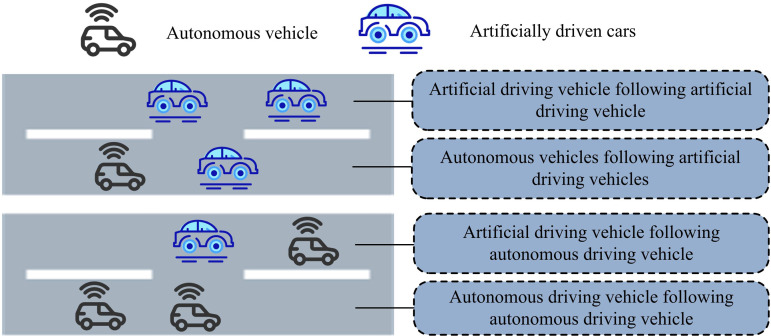
Schematic diagram of vehicle following mode in mixed roads.

In Equation (5), $q_{e}$ denotes the flow rate in steady TF state. $v_{e}$ denotes the vehicle speed in steady TF condition. $s_{e}$ denotes the headway spacing in steady TF condition. $v_{z}$ denotes the free flow speed. $s_{0}$ denotes the headway at stopping, and $s_{0}$ is taken as 7m for the study. *T* denotes the average safe headway. The schematic diagrams (SDs) of different vehicle following modes in a mixed roadway are shown in Fig 2.

In Fig 2, there are four vehicle following modes, autonomous driving vehicle (AuDV) following AuDV, AuDV following artificially driven vehicle (ArDV), ArDV following AuDV, and ArDV following ArDV, in the road with mixed AuDV and ArDV traffic. The headway distances are $T_{cc}$, $T_{ch}$, $T_{hc}$, and $T_{hh}$, respectively. Assuming that the percentage of AuDVs is *p*, the average headway distance *T* is calculated as shown in Equation (6) when there is no dedicated lane for CVs.


$$T=p^{2}T_{cc}+p(1-p)T_{ch}+p(1-p)T_{hc}+{\left(1-p\right)}^{2}T_{hh}$$ (6)

In a conventional road system without dedicated lanes for CVs, the road resistance function $t_{a}^{M}$ is shown in Equation (7).


$$t_{a}^{M}=t_{a}^{z}\left[1+\alpha {\left(\frac{x_{a}}{kC_{a}^{M}}\right)}^{\beta }\right]$$ (7)

**Fig 3:**
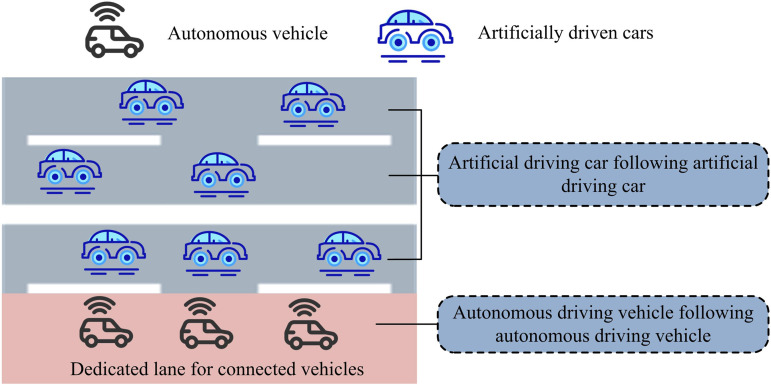
Schematic diagram of following mode after setting up dedicated lanes.

In Equation (7), *k* denotes the number of lanes in roadway segment *a*. $C_{a}^{M}$ denotes the capacity of the mixed lane. Fig 3 depicts the SD of the potential subsequent mode if the designated lane for CVs is enabled.

In Fig 3, when the dedicated lane for CVs is set, there are only two following modes in the road system, i.e., AuDV following AuDV and ArDV following ArDV. The road resistance function $t_{a}^{D}$ for lane for CVs road segment *a* is shown in Equation (8).


$$t_{a}^{D}=t_{a}^{z}\left[1+\alpha {\left(\frac{x_{a}p}{C_{a}^{D}}\right)}^{\beta }\right]$$ (8)

In Equation (8), $t_{a}^{D}$ denotes the capacity of lane for CVs. The road resistance function $t_{a}^{N}$ for the remaining conventional lanes is shown in Equation (9).


$$t_{a}^{N}=t_{a}^{z}\left[1+\alpha {\left(\frac{x_{a}(1-p)}{(k-1)C_{a}^{N}}\right)}^{\beta }\right]$$ (9)

In Equation (9), $C_{a}^{N}$ denotes the capacity of regular lanes. In summary, the OF of the LLPM is shown in Equation (10).


$$\min Z(x)=\sum_{a\in A_{M}}\int_{0}^{x_{a}}t_{a}^{M}(x)dx+\sum_{a\in A_{D}}\int_{0}^{x_{a}p}t_{a}^{N}(x)dx+\sum_{a\in A_{D}}\int_{0}^{x_{a}(1-P)}t_{a}^{D}(x)dx$$ (10)

In Equation (10), $A_{M}$ denotes the set of road segments without dedicated lanes. $A_{D}$ denotes the set of road segments with dedicated lanes. In summary, in the dual layer planning model for urban connected vehicle lanes constructed in the research, the upper layer planning model takes minimizing the total travel cost within the TS as the planning objective, with constraints including traffic fairness and construction costs, and also considers the influence of the lower layer model. The lower level planning model solves the traffic allocation problem in the TS under the constraints of the upper level planning model, and then predicts the traffic volume on each road. There is a close relationship between lane planning and traffic accidents. Reasonable lane planning can not only improve road capacity, but also effectively reduce the occurrence of traffic accidents [26,27].

### 2.2. Lane programming model solving based on improved FW algorithm

To further solve the BLPM constructed in Section 2.1, the study analyzes the algorithms for solving the upper and lower level models separately. The upper-level model in the lane planning for urban CVs BLPM is used to solve the decision variables. When faced with a complex road system, the problem has a higher dimension. Heuristic algorithms are a class of algorithms that utilize rules of thumb and heuristic information for searching in solving complex problems. Their ability to provide feasible solutions to the combinatorial optimization problem to be solved at an acceptable cost has significant advantages when dealing with high dimensional problems. Therefore, the study solves the higher model using WOA. The hunting behavior of humpback whale groups in the wild is mimicked by the meta-heuristic optimization algorithm WOA. By mimicking the whale group’s self-organization and self-adaptation, it determines the best course of action [28]. The encirclement strategy of WOA is shown in Equation (11).


$$\{\begin{array}{c} D=\left|CX^{*}\left(t\right)-X\left(t\right)\right|\\ X\left(t+1\right)=X^{*}\left(t\right)-AD \end{array}$$ (11)

**Fig 4:**
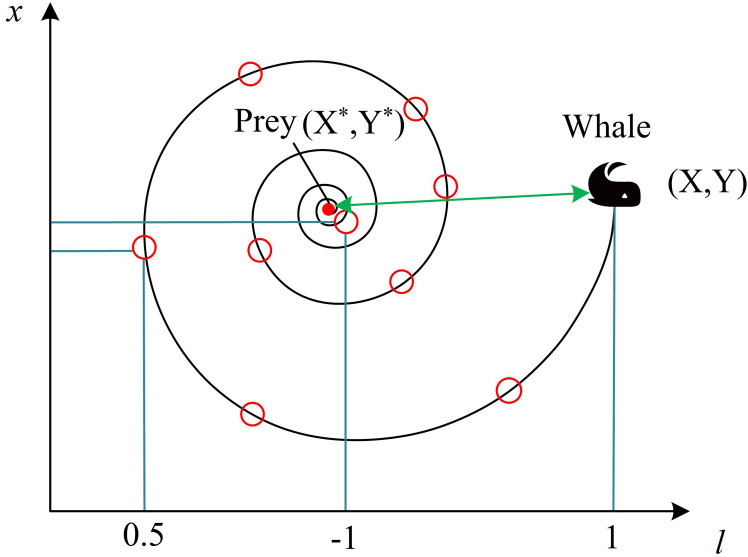
Schematic diagram of spiral update.

In Equation (11), *D* is the distance between the individual’s location and the optimal location at iteration *t* times. $X^{*}\left(t\right)$ is the position of the prey. $X\left(t\right)$ denotes the current position of the whale. $X\left(t+1\right)$ is the position of the whale at the next moment. *C* and *A* are two coefficient vectors. The bubble net attack of WOA simulates the hunting behavior of humpback whales, including spiral update (SU) and shrink wrap (SW) [29]. The SU mimics the way a feeding humpback whale would swim in a spiral toward its meal. First, the distance between the position of the whale and the prey is computed, and then a spiral equation is developed to replicate the spiral motion of the humpback whale. In Fig 4, the schematic is displayed.

**Fig 5:**
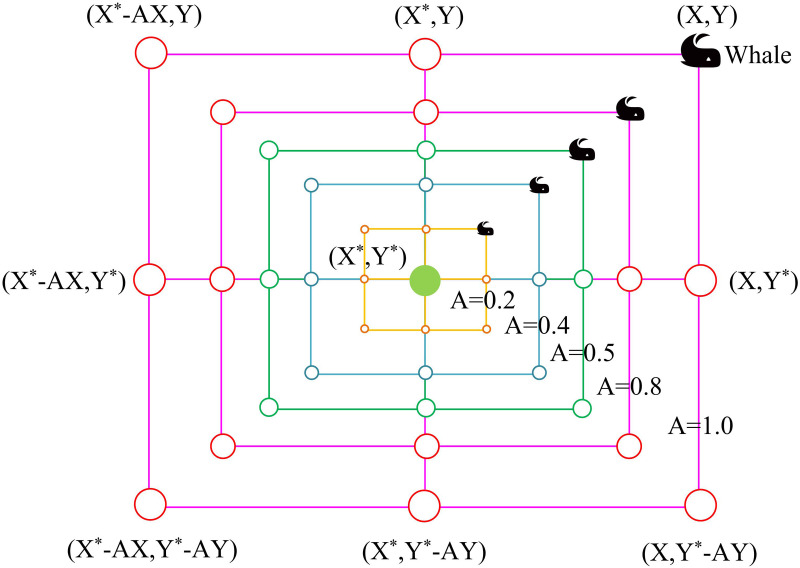
Schematic diagram of shrink wrap.

The SW behavior simulates the behavior of humpback whales that gradually encircle their prey during feeding attacks. The new individual position vector shrinks to the optimal whale individual position vector, which helps the whale population to approach the global optimal solution [30]. The SD of SW is shown in Fig 5.

The calculation of SU and SW is shown in Equation (12).


$$\{\begin{array}{c} X\left(t+1\right)=D^{'}e^{bl}\cos(2\pi )+X^{*}\left(t\right)\\ D^{'}=\left|X^{*}\left(t\right)-X\left(t\right)\right| \end{array}$$ (12)

In Equation (12), *b* controls the shape of the spiral. *l* denotes the random number. Whales randomly choose positions from the existing population to conduct position updates and hunt for prey during the random search phase of WOA. This helps prevent the algorithm from settling on a locally optimal solution. The random search phase is shown in Equation (13).


$$\{\begin{array}{c} D=\left|-X\left(t\right)+CX_{rand}\left(t\right)\right|\\ X\left(t+1\right)=-AD+X_{rand}\left(t\right) \end{array}$$ (13)

In Equation (13), $X_{rand}\left(t\right)$ denotes the random location of the prey. However, WOA searches for new solutions by random numbers, which may fall into one end of binary variables in lane programming model solving. Therefore, the study uses logical operations to fully search the solution space. The function updates the logical expression as shown in Equation (14).


$$X(n+1)=X(n)⊕B\left(X(n)⊕X_{rand}(n)\right)$$ (14)

In Equation (14), X(n+1) denotes the update solution. X(n) denotes the current solution. *B* denotes the parameter. X(n) denotes the randomly generated solution.The FW algorithm is used in the study to solve the lower model. A traditional technique for resolving nonlinearly restricted optimization issues is the FW algorithm. It obtains feasible descent directions by linearly approximating the OF and performs a one-dimensional search in these directions to gradually approximate the optimal solution [31,32]. For problem minf(x), assuming that there is a linear function g(x) and the OF f(x) is differentiable, the expansion of the OF at $x_{k}$ is shown in Equation (15).


$$f(x)\approx f(x_{k})+\nabla f{\left(x_{k}\right)}^{T}(x-x_{k})$$ (15)

**Fig 6:**
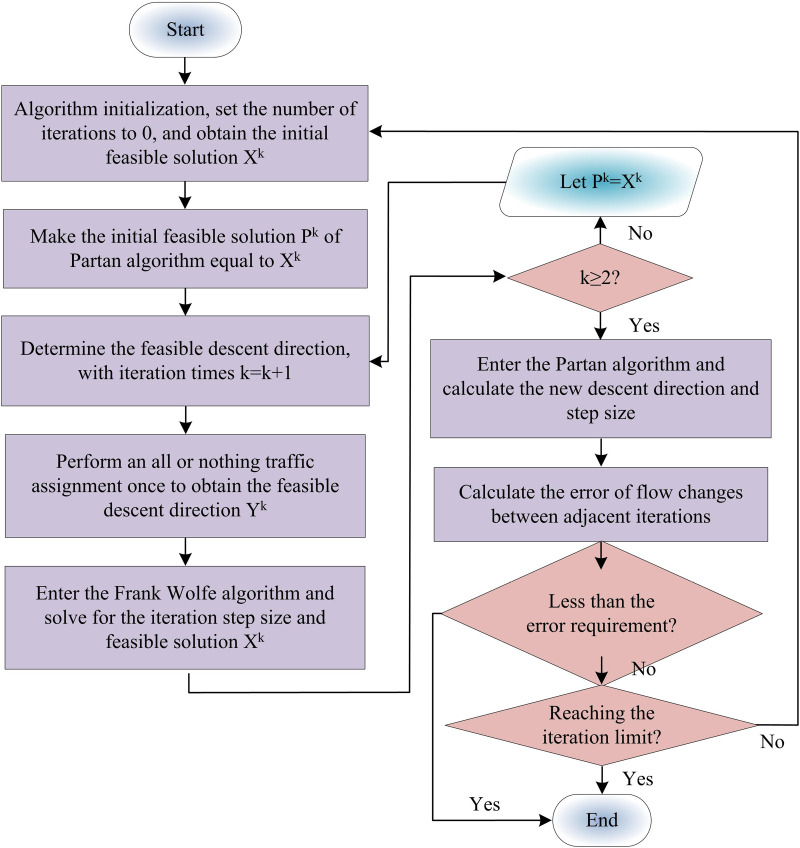
Process diagram for improving Frank Wolfe algorithm.

In Equation (15), minf(x) can be converted to a linear programming problem $\min\nabla f{\left(x_{k}\right)}^{T}x_{k}$ since both $f(x_{k})$ and $\nabla f{\left(x_{k}\right)}^{T}x_{k}$ are constants. However, the traditional FW algorithm converges slowly in the late iteration stage, and also suffers from significant oscillations. Therefore, the study uses Partan algorithm to optimize the FW algorithm [33]. Partan’s algorithm is a computer-implemented algorithm for optimization problems and is particularly suitable for maximum likelihood estimation of partially linear models. Fig 6 depicts the flow of the enhanced FW algorithm.

Among them, the initial feasible solution $X^{k}$ of the FW algorithm is calculated as shown in Equation (16).


$$X^{k}=P^{k}+\lambda^{k}(Y^{k}-P^{k-1})$$ (16)

In Equation (16), $P^{k}$ denotes the initial feasible solution of Partan algorithm. $\lambda^{k}$ denotes the optimal SS, which takes the value in the range of [0,1]. $Y^{k}$ denotes the feasible descent direction. The calculation of $P^{k}$ is shown in Equation (17).


$$P^{k}=X^{k}+\eta^{k}(P^{k-2}-X^{k})$$ (17)

In Equation (17), *k* denotes the iterations. $\eta^{k}$ denotes the optimal SS. Given $\eta^{1}=0$, the minimum SS $\eta_{\min}^{k}$ is calculated as shown in Equation (18).


$$\eta_{\min}^{k}= \{\begin{array}{c} 1+\frac{1}{\left(1-\lambda^{k}\right)\left(1-\lambda^{k-1}\right)\left[\left(1-\eta^{k-1}\right)/\eta_{\min}^{k-1}\right]-1},if\eta^{k-1}<0\\ 1+\frac{1}{\left(1-\lambda^{k}\right)\left(1-\lambda^{k-1}\right)(1-\eta^{k-1})-1},if\eta^{k-1}\geq 0 \end{array}$$ (18)

The iteration error *E* is calculated as shown in Equation (19).


$$E=\frac{\sum_{a}\left|p^{k}-p^{k-1}\right|}{\sum_{a}p^{k}}$$ (19)

**Fig 7:**
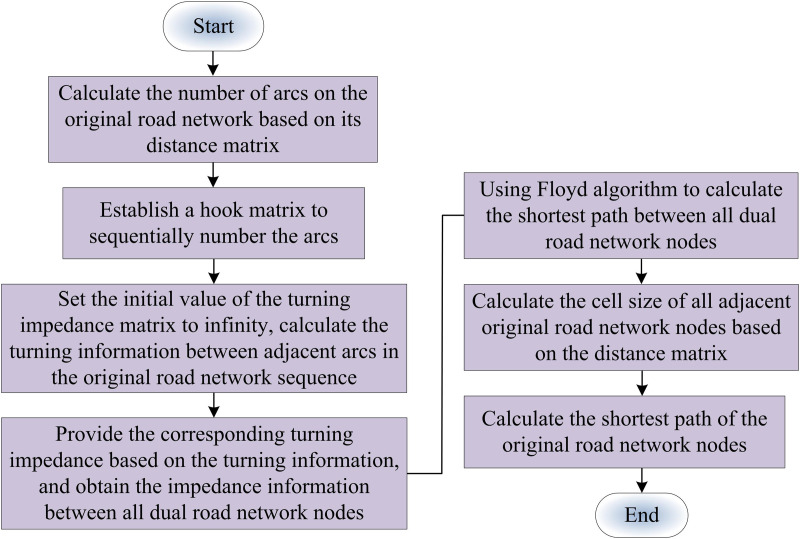
Flowchart of Floyd’s shortest path dual graph algorithm.

In Equation (19), $p^{k}$ and $p^{k-1}$ denote the flow rate of each road segment at the end of the *k* th and k−1 th iterations, respectively. The improved FW algorithm solves for the feasible descent direction by shortest path generation. In contrast, in the lane planning for urban CVs model, intersection steering impedance must be considered when finding the shortest path. Therefore, it is studied to convert the original road network into dual road network with the help of dyadic graph idea. The generic shortest path approach can be used to find the shortest path between any two nodes in a dual road network, which is a road network map without turn impedance. Floyd’s approach finds the shortest path between numerous source points in a given weighted network by utilizing the concept of dynamic programming. It has been widely utilized in dense maps and is capable of handling the directed graph shortest path problem appropriately [34,35]. The study suggests a Floyd shortest path dyadic network algorithm in light of this. The specific computational flow is shown in Fig 7.

Fig 7 shows the shortest path calculation of the original road network nodes. Firstly, according to the original road network start and end numbers, the set of arcs with the start number as the arc head and the set of arcs with the end number as the arc tail are found based on the neighboring original road network node tuples, respectively. Second, find the corresponding set of dual road network nodes numbers in the hook matrix. The shortest dual road network nodes paths are found by traversing the set of dual road network nodes whose start numbers are the first arcs and the set of dual road network nodes whose end numbers are the last arcs, respectively. Finally, the dual road network nodes paths are substituted into the hook matrix to obtain the corresponding shortest paths of the original road network nodes.

## 3. Results

The study builds a lane planning for urban CVs BLPM and uses the improved WOA and FW algorithms to solve the model. However, its feasibility has to be further verified. The study primarily examines the enhanced WOA and enhanced FW algorithms’ performances from two angles. The correctness and feasibility of the algorithms and models are then confirmed by analyzing arithmetic situations in various circumstances.

### 3.1. Performance analysis of the solving algorithm

**Fig 8:**
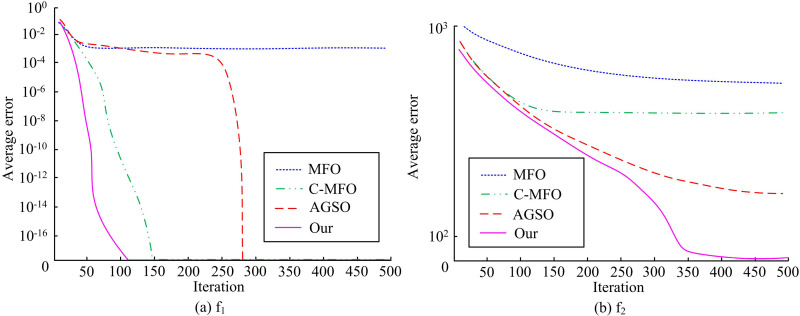
Test function convergence curve.

To investigate the effectiveness of the proposed improved WOA algorithm, the study selects two benchmark test functions, f1 and f2, for the experiment, i.e., $f1(x)=\frac{-0.5+\sin^{2}(-x_{2}^{2}+x_{1}^{2})}{{\left[0.001(x_{2}^{2}+x_{1}^{2})+1\right]}^{2}}+0.5$ and $f2(x)=\sum_{i=1}^{d}\left[+10-10\cos(2\pi xi)+x_{i}^{2}\right]$, with the optimal value of 0, on the MATLAB 2020 simulation and experimentation platform. The study sets the dimensions to 30, the maximum iterations to 500, and the epoch to 30. The proposed improved WOA algorithm is compared with the moth-flame optimization (MFO) algorithm, the MFO algorithm incorporating chaotic strategies (C-MFO) and adaptive glowworm swarm optimization (AGSO). Fig 8 presents the findings. In Fig 8(a), compared to the other three algorithms, the study mentions that the improved WOA algorithm performs best on the f1 test function. It converges quickly after the start of the iterations and finds the optimal solution 0 when the iterations is about 110. The next best algorithm is the C-MFO algorithm, which finds the optimal solution 0 when the iterations is about 150. The MFO algorithm fails to solve the optimal solution. In Fig 8(b), the study mentions that the improved WOA algorithm still performs the best on the f2 test function, converging at a number of iterations of 350. When the number of iterations is less than 300, the improved WOA algorithm can quickly locate the potential optimal solution area in the search space, and the average error decreases rapidly. As the iteration progresses, the improved WOA algorithm gradually approaches the global optimal solution, and the gradient changes in the search space are small, resulting in an average error that tends to flatten out when the number of iterations is around 350. Although the convergence speed has slowed down compared to the F1 test function, convergence is still achieved in a relatively small number of iterations. It is followed by AGSO algorithm. The findings indicate that the study’s enhanced WOA algorithm performs better during optimization searches and has a faster rate of convergence, suggesting its viability and efficacy.

**Fig 9:**
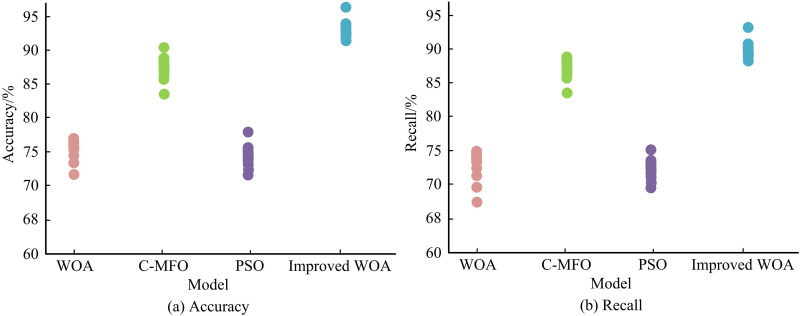
Comparison of accuracy and recall of four algorithms.

The Iris dataset is used in the study’s testing to further examine the optimality seeking performance of the suggested improved WOA algorithm. The traditional WOA algorithm, the C-MFO algorithm, and the particle swarm optimization (PSO) algorithm are compared for accuracy and recall with the modified WOA algorithm. Fig 9 presents the findings. The accuracy of the suggested enhanced WOA method is 95.27% on the Iris dataset, as shown in Fig 9(a), which is much higher than the other three algorithms. The C-MFO algorithm, which has an accuracy of 90.14%, comes next. The suggested enhanced WOA algorithm continues to have the maximum recall of 92.65% in Fig 9(b). The findings indicate that the suggested enhanced WOA algorithm performs optimization searching more effectively and with a high degree of accuracy.

**Fig 10:**
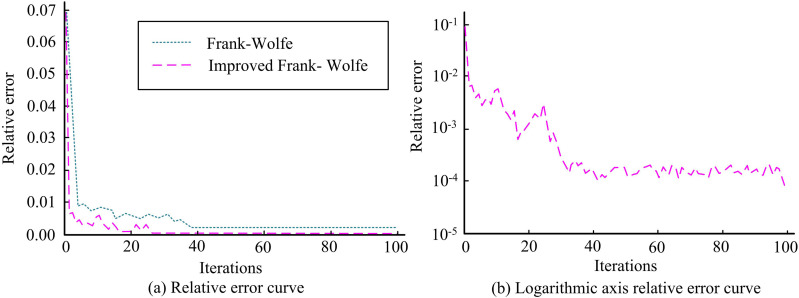
Relative error iteration curve.

The research employs numerical experiments on a road network with 21 nodes and 48 road segments to examine the performance of the modified FW algorithm. All road segments have four lanes, and 50% of vehicles are expected to use them. Fig 10 displays the relative error iteration curves for both the conventional FW algorithm and the enhanced FW technique. In Fig 10(a), the study’s revised FW algorithm has a faster rate of convergence than the conventional FW algorithm. When there have been roughly thirty iterations and the relative error is smaller, it converges. To more clearly illustrate the convergence effect of the modified FW method, the study logarithmically scales the relative error axis, as illustrated in Fig 10(b). The enhanced FW algorithm has a convergence limit of about 10^-4^. The findings demonstrate the viability of the study’s development plan and its ability to significantly increase the FW algorithm’s convergence speed and accuracy.

### 3.2. Case analysis

**Fig 11:**
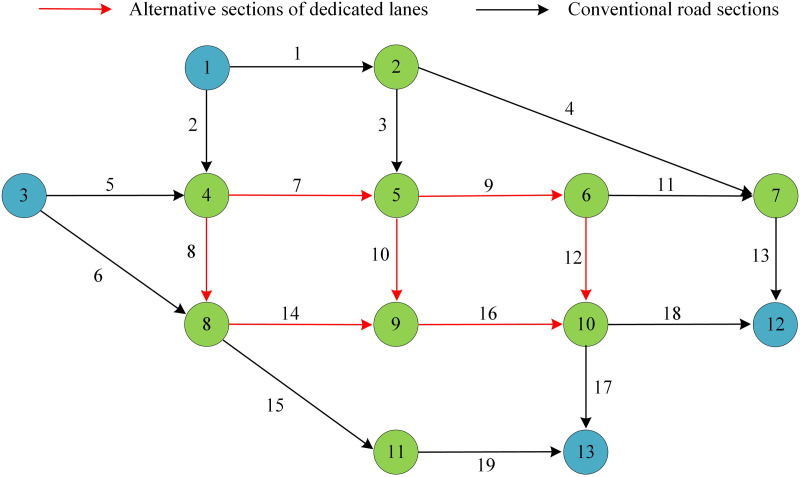
Nguyen Dupuis Network.

The study is conducted in MATLAB 2020 environment for experiments. The proposed lane programming model is applied to the Nguyen-Dupuis network consisting of 13 nodes and 19 road segments. The 13 nodes of the network are numbered from 1 to 13, representing key intersections or transportation hubs in the transportation network. The length, free flow time, and capacity of the road section are all set based on the raw data of the Nguyen Dupuis network [36]. The Nguyen Dupuis network is shown in Fig 11.

The study uses nodes 1 and 3 as the starting point and nodes 12 and 13 as the end point, with a travel demand of 800 pcu/h starting at node 1 and 600 pcu/h starting at node 3. It is assumed that the PR of CVs are all 50% and that there are seven dedicated lane alternatives in the network (sections 7, 8, 9, 10, 12, 14 and 16). Table 1 displays the Nguyen-Dupuis network’s specific parameters.

**Table 1:** Specific parameters of Nguyen-Dupuis network.

Road section number	Number of lanes	Road length	Free flowing driving time/min
1	3	3.6	9
2	4	3.6	7
3	3	3.6	7
4	2	10.8	14
5	3	3.6	9
6	3	7.2	12
7	4	3.6	3
8	3	3.6	9
9	3	3.6	5
10	3	3.6	13
11	3	3.6	2
12	4	3.6	9
13	3	3.6	9
14	4	3.6	10
15	2	7.2	9
16	4	3.6	6
17	3	3.6	5
18	4	3.6	9
19	3	3.6	11

**Fig 12:**
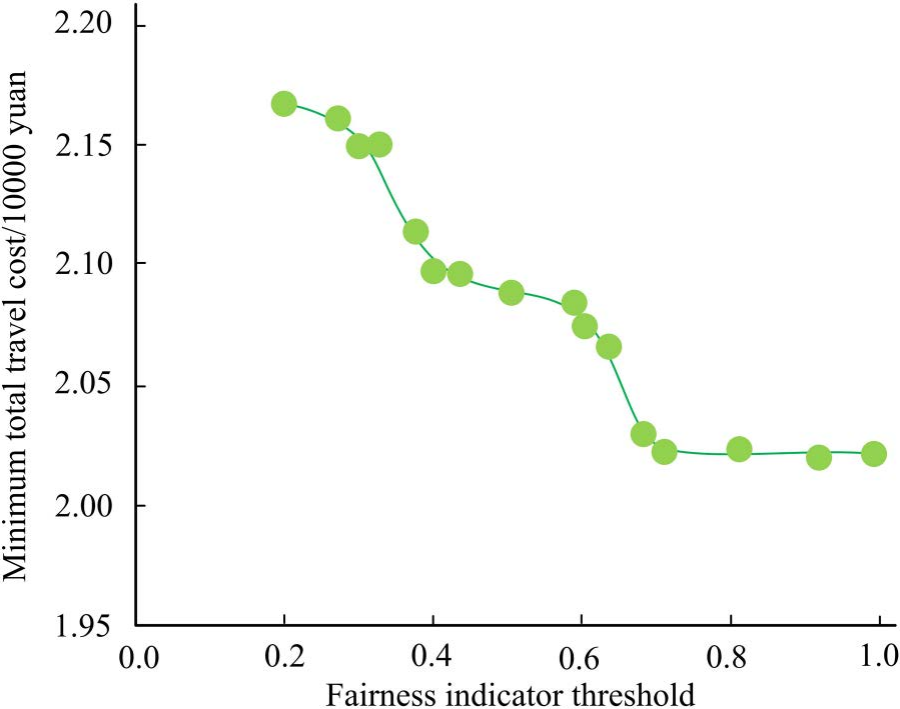
Fairness index threshold on the total travel cost.

To analyze the impacts of dedicated lane deployment on the roadway network, the study conducts a comparative analysis in two scenarios, deployment and no deployment. The Federal Highway Administration of the United States conducted traffic surveys on a large number of road sections and conducted regression analysis. It is recommended to use two parameter values, α=0.15 and β=4, in the BPR function, which can better reflect the impedance characteristics of road sections under general traffic conditions. Therefore, the study sets *α* to 0.15, *β* to 4 [23]. Set the budget for dedicated lane deployment to 230,000 yuan, and the construction cost per unit length of dedicated lane for CVs to 8760 yuan. The study established a 7-meter parking headway, a 60km/h free flow speed, and a 50% CV percentage. Fig 12 displays the road system’s minimum TTC curve in respect to the fairness indicator’s threshold. As the threshold value rises, the road system’s minimum TTC progressively falls. At 20.2 thousand dollars, the lowest TTC of the system is reached when the threshold value approaches 0.7. The minimum TTC of the system does not change if the transportation equity index threshold continues to increase. This may be because the current road system resource allocation has reached its limit, and it is not possible to further optimize travel costs by improving traffic fairness. The results indicate that improving traffic equity within a certain range can help reduce overall travel costs. In urban transportation planning and policy-making, reasonable thresholds for traffic equity indicators should be set to achieve the best balance between travel costs and traffic equity.

The optimal deployment scheme under different transportation fairness indicator thresholds is shown in Table 2. When the threshold value of traffic fairness index is 0.2, the TTC is 21.7 million yuan. Moreover, under different traffic fairness index thresholds, the lane planning for urban CVs model constructed by the study is able to give the corresponding lane planning scheme, which has certain feasibility and effectiveness.

**Table 2:** The optimal deployment plan under different thresholds of traffic fairness indicators.

Threshold	Alternative sections of dedicated lanes							Total cost/10000 yuan
	16	14	12	10	9	8	7	
0.2	0	1	1	1	0	1	0	2.17
0.3	0	0	1	1	0	0	1	2.16
0.4	1	0	1	1	0	1	0	2.10
0.5	0	0	1	1	1	1	0	2.09
0.6	1	0	1	0	0	1	1	2.08
0.7	1	1	1	1	1	1	0	2.02

**Fig 13:**
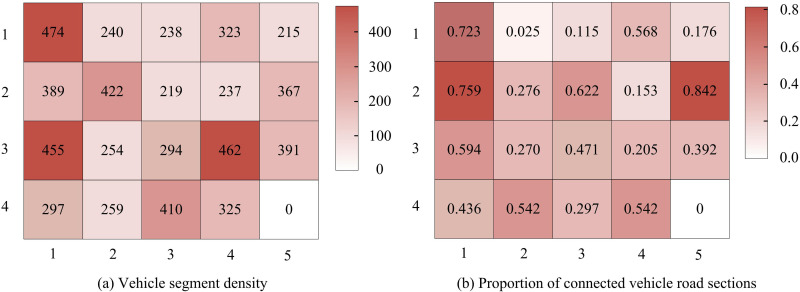
Road density and connected vehicle road sections.

The heat map of vehicle section density and the heat map of the proportion of CVs sections for the road sections in the equilibrium state are shown in Fig 13. The road sections in the Nguyen-Dupuis network have the situation that some sections are overcrowded while some sections have less traffic. Moreover, there is no obvious relationship between the proportion of CVs roadway segments and the density of vehicle roadway segments.

**Fig 14:**
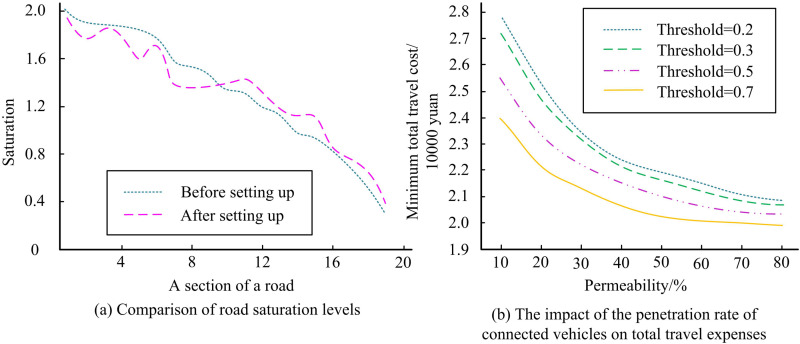
Road saturation and total travel cost.

To explore the effectiveness of the proposed lane planning for urban CVs model, the study compares the ROAD saturation before and after the deployment. The TTC is also analyzed after the installation of the dedicated lanes. The comparison of road saturation before and after the deployment of dedicated lanes and the effect of the PR of CVs on the TTC are shown in Fig 14. In Fig 14(a), the lane planning for urban CVs model constructed by the study optimizes the road network by balancing the passenger flow between congested and relaxed road sections. In Fig 14(b), the TTC of the road system decreases gradually with the increase of the PR of CVs at different thresholds. This may be because CVs, through real-time information sharing and collaborative decision-making, can reduce congestion and delays, thereby lowering travel costs. When the threshold value is 0.7 and the PR of CVs is 80%, the TTC is the lowest, which is 20.2 million yuan. The results show that at higher PRs of CVs, the lane planning for urban CVs model constructed in the study can still deduce the TTC while taking into account the fairness.

## 4. Discussion and conclusion

To address the safety hazards caused by the mixing of CVs and ordinary manually-driven vehicles, the study considered providing dedicated lanes for CVs in the roadway, and constructed a lane planning for urban CVs BLPM. An improved WOA and FW algorithm were proposed to solve the model. The results indicated that the improved WOA algorithm proposed by the study performed best on the f1 test function. It converged quickly after the start of the iterations and found the optimal solution 0 at about 110 iterations. The improved WOA algorithm still performed best on the f2 test function and converged at 350 iterations. The accuracy of the improved WOA algorithm on the Iris dataset was 95.27%. The recall of the improved WOA algorithm was 92.65%. Compared to the traditional FW algorithm, the improved FW algorithm converges faster. It can converge when the iterations is about 30, and the relative error is lower, with a convergence limit of about 10^-4^. When the threshold value of the transportation fairness index reaches 0.7, the lowest TTC of the system is at least 20,200 yuan. If the transportation fairness index threshold continues to increase, the lowest TTC of the system does not change. Compared to the traditional FW algorithm, the improved FW algorithm converges faster. It could converge when the number of iterations was about 30 times, and the relative error was lower, with its convergence limit around 10^-4^. When the threshold value of the transportation fairness index reached 0.7, the lowest TTC of the system was 20.2 million yuan. If the transportation fairness index threshold continues to increase, the lowest TTC of the system does not change. The research concludes that: 1. Logical operations can effectively improve the optimization performance of WOA algorithm. 2. The Partan algorithm can effectively improve the convergence speed and accuracy of the Frank Wolfe algorithm. Within a certain range, improving transportation fairness can help reduce overall travel costs. The total travel cost of the road system gradually decreases with the increase of the penetration rate of CVs.

To sum up, the experimental outcomes validate the practicality and efficiency of the suggested lane planning strategy for urban CVs BLPM and solution method. The improved WOA and Frank Wolfe algorithms demonstrated good optimization ability and fast convergence speed in experiments, which means that the proposed algorithm can effectively find the optimal or near optimal lane planning scheme in complex transportation networks in a short time. In practical traffic management, by utilizing improved WOA and Frank Wolfe algorithms to optimize lane planning schemes, more efficient lane planning schemes can be designed, transforming the algorithm’s optimization ability into more efficient traffic flow allocation, reducing congestion, and improving road utilization efficiency. In addition, the relatively low relative error of the algorithm means that the difference between the predicted results and the actual traffic conditions is small, which can reduce traffic control errors caused by inaccurate predictions and improve the effectiveness of traffic management measures. Applying the proposed dual layer planning model for urban connected vehicle lanes to mixed driving traffic flow can help reduce the complexity of mixed traffic flow, minimize the mutual influence between CVs and traditional manual driving vehicles, and enhance the market penetration rate of CVs. However, in order to simplify the calculation, the study only considered the case of CVs mixed with ordinary cars, and there are more vehicles such as buses and non-motorized vehicles in the actual road. Therefore, the relationship between CVs and buses and non-motorized vehicles should be further explored in future studies to provide more comprehensive suggestions for urban road construction and management.

## Supporting information

S1 File Minimal data set definition. (DOCX)
