# Supplementary material for: Urban connected vehicle lane planning based on improved Frank Wolfe algorithm
Source: PLoS One. 2025 Apr 22;20(4):e0321540. doi: 10.1371/journal.pone.0321540 (PMC12013868; doi:10.1371/journal.pone.0321540)
Supplement: S1 File — (DOCX) [file pone.0321540.s001.docx]

**Minimal Data Set Definition**

**Fig.8 Test function convergence curve**

| Tests | Algorithm | Iteration | | | | | | | | | |
| --- | --- | --- | --- | --- | --- | --- | --- | --- | --- | --- | --- |
|  |  | 50 | 100 | 150 | 200 | 250 | 300 | 350 | 400 | 450 | 500 |
| f1 | MFO | 10^-1.0^ | 10^-3.1^ | 10^-3.1^ | 10^-3.1^ | 10^-3.1^ | 10^-3.1^ | 10^-3.1^ | 10^-3.1^ | 10^-3.1^ | 10^-3.1^ |
|  | C-MFO | 10^-1.0^ | 10^-10.7^ | 0 | 0 | 0 | 0 | 0 | 0 | 0 | 0 |
|  | AGSO | 10^-1.0^ | 10^-3.0^ | 10^-3.5^ | 10^-3.6^ | 10^-4.1^ | 0 | 0 | 0 | 0 | 0 |
|  | Our | 10^-1.0^ | 10^-17.3^ | 0 | 0 | 0 | 0 | 0 | 0 | 0 | 0 |
| f2 | MFO | 10^2.92^ | 10^2.88^ | 10^2.80^ | 10^2.77^ | 10^2.75^ | 10^2.72^ | 10^2.71^ | 10^2.70^ | 10^2.69^ | 10^2.68^ |
|  | C-MFO | 10^2.74^ | 10^2.62^ | 10^2.60^ | 10^2.60^ | 10^2.59^ | 10^2.59^ | 10^2.59^ | 10^2.59^ | 10^2.59^ | 10^2.59^ |
|  | AGSO | 10^2.74^ | 10^2.61^ | 10^2.45^ | 10^2.39^ | 10^2.34^ | 10^2.27^ | 10^2.25^ | 10^2.22^ | 10^2.20^ | 10^2.19^ |
|  | Our | 10^2.72^ | 10^2.59^ | 10^2.43^ | 10^2.36^ | 10^2.30^ | 10^2.16^ | 10^0.73^ | 10^0.33^ | 10^0.30^ | 10^0.31^ |

**Fig.9 Comparison of Accuracy and Recall of Four Algorithms**

| Index | Models | | | |
| --- | --- | --- | --- | --- |
|  | WOA | C-MFO | PSO | Improved WOA |
| Accuracy/% | 77.04 | 90.14 | 76.62 | 95.27 |
| Recall/% | 74.95 | 89.46 | 74.17 | 92.65 |

**Fig.10 Relative error iteration curve**

| Index | | Iteration | | | | | | | | |
| --- | --- | --- | --- | --- | --- | --- | --- | --- | --- | --- |
|  |  | 20 | | 40 | | 60 | | 80 | | 100 |
| Relative error | Frank-Wolfe | 0.0057 | 0.0210 | | 0.0210 | | 0.0210 | | 0.0210 | |
|  | Improved Frank-Wolfe | 0.0137 | 0.0001 | | 0.0001 | | 0.0001 | | 0.0001 | |
| Logarithmic axis relative error | | 10^-2.9^ | 10^-3.8^ | | 10^-4.1^ | | 10^-3.8^ | | 10^-4.2^ | |

**Fig.12 The impact of fairness index threshold on the total travel cost of the system**

| Index | Fairness indicator threshold | | | | |
| --- | --- | --- | --- | --- | --- |
|  | 0.2 | 0.4 | 0.6 | 0.8 | 1.0 |
| Minimum total travel cost/10000 yuan | 2.166 | 2.101 | 2.077 | 2.073 | 2.073 |

**Fig.14 Road saturation and total travel cost**

**(a) Comparison of road saturation levels**

| Saturation | A section of a road | | | | |
| --- | --- | --- | --- | --- | --- |
|  | 4 | 8 | 12 | 16 | 20 |
| Before setting up | 1.86 | 1.54 | 1.20 | 0.81 | 0.20 |
| After setting up | 1.77 | 1.35 | 1.30 | 0.85 | 0.28 |

**(b) The impact of the penetration rate of connected vehicles on total travel expenses**

**(10000 yuan)**

| Threshold | Permeability/% | | | | | | | |
| --- | --- | --- | --- | --- | --- | --- | --- | --- |
|  | 10 | 20 | 30 | 40 | 50 | 60 | 70 | 80 |
| Threshold=0.2 | 2.77 | 2.53 | 2.35 | 2.24 | 2.19 | 2.15 | 2.11 | 2.08 |
| Threshold=0.3 | 2.72 | 2.47 | 2.32 | 2.22 | 2.17 | 2.12 | 2.08 | 2.06 |
| Threshold=0.5 | 2.54 | 2.33 | 2.22 | 2.15 | 2.10 | 2.07 | 2.04 | 2.04 |
| Threshold=0.7 | 2.40 | 2.21 | 2.13 | 2.07 | 2.02 | 2.01 | 2.00 | 1.99 |
